# Ultrasonic Cavitation Erosion Behavior of GX40CrNiSi25-20 Cast Stainless Steel through Yb-YAG Surface Remelting

**DOI:** 10.3390/ma17174180

**Published:** 2024-08-23

**Authors:** Daniela Cosma, Ion Mitelea, Ilare Bordeașu, Ion Dragoș Uțu, Corneliu Marius Crăciunescu

**Affiliations:** 1 Department of Materials and Fabrication Engineering, Politehnica University Timisoara, Bulevardul Mihai Viteazul nr. 1, 300222 Timișoara, Romania; daniela.alexa@fih.upt.ro (D.C.); ion.mitelea@upt.ro (I.M.); corneliu.craciunescu@upt.ro (C.M.C.); 2 Department of Mechanical Machines, Equipment and Transports, Politehnica University Timisoara, Bulevardul Mihai Viteazul nr. 1, 300222 Timișoara, Romania; ilare.bordeasu@upt.ro

**Keywords:** laser remelting Yb-YAG, cast stainless steel, cavitation erosion, microstructure

## Abstract

Laser beam remelting is a relatively simple and highly effective technique for the physical modification of surfaces to improve resistance to cavitation erosion. In this study, we investigated the effect of laser remelting on the surface of cast stainless steel with 0.40% C, 25% Cr, 20% Ni, and 1.5% Si on cavitation erosion behavior in tap water. The investigation was conducted using a piezoceramic crystal vibrator apparatus. Base laser beam parameters were carefully selected to result in a defect-free surface (no porosity, material burn, cracks) with hardness capable of generating better resistance to cavitation erosion. The experimental results were compared with those of the reference material. Surface morphology and microstructure evolution after cavitation tests were analyzed using an optical metallographic microscope (OM), scanning electron microscope (SEM), and hardness tests to explore the mechanism of improving surface degradation resistance. The conducted research demonstrated that surfaces modified by laser remelting exhibit a 4.8–5.1 times greater increase in cavitation erosion resistance due to the homogenization of chemical composition and refinement of the microstructure, while maintaining the properties of the base material.

## 1. Introduction

Cavitation erosion is induced by the impact of bubbles formed because of a sudden drop in the local pressure of a liquid near the surface of mechanical system components. The implosion of these bubbles can release shock waves and microjets, reaching speeds of 300 to 500 m/s [1,2,3]. The repeated impact of microjets and shock waves on the surface leads to plastic deformation, mechanical crushing, the formation of microcracks, and material loss, causing pronounced wear and potential damage to components [4,5,6]. The growth and degradation time of cavitation bubbles occur within milliseconds, and the microcurrents generated during their impulse apply pressures to the solid surface ranging from 1000 to 4000 MPa, exceeding the yield strength of most materials [2,7,8,9].

Currently, various methods are under development to modify the surfaces of metals and metal alloys to enhance resistance to cavitation erosion. These methods include thermal spraying, thermochemical nitriding treatment, welding overlay, surface hardening through induction or high-energy beam processes, and local re-melting with the TIG electric arc, among others [1,2,3,4,5,6,7,8,9,10,11,12,13,14,15,16,17,18,19,20]. For example, Wang Jiewen et al. [16] demonstrated that the amorphous structure composite from Fe-based alloys deposited by HVOF thermal spraying provides remarkable protection against cavitation erosion. Thermal spraying techniques are attractive due to their ability to produce coatings with a high deposition rate and minimal modification of substrate properties, thanks to the low thermal shock intensity of the process. However, the existence of porosities, poor substrate adherence, and non-homogeneous microstructure limit the cavitation erosion resistance of deposited layers [15,16].

Some authors [17,18,19,20] have shown that applying thermochemical nitriding treatment to both Ti and its biphasic alloys (Ti–6Al–4V) significantly improves cavitation resistance. On the other hand, Espitia et al. [18] found less favorable effects of nitriding when applied to stainless steels with martensitic microstructure. The initial microstructure of the nitrided material, the existence of the chemical combination zone, and the kind of nitrides that developed in the layer are some of the possible causes of these disparate impacts of nitriding on cavitation erosion resistance.

Welding overlay techniques with the electric arc [21], while enhancing resistance to cavitation erosion, have the disadvantage of inducing undesirable microstructural changes in the substrate material due to the high thermal shock. Consequently, it is often necessary to perform subsequent heat treatment after the welding operation.

The TIG local surface remelting process of engineering alloys, although slower compared to other electric arc welding procedures, presents several advantages, such as lower requirements for linear energy, reduced residual stresses and deformations, fine finishing of the grain structure and microstructure of the fusion zone, and a less extensive heat-affected zone [22,23]. Technological variations of surface treatment with laser beams (local remelting, hardening through martensitic quenching, alloying, etc.) contribute to improving the mechanical properties and cavitation erosion resistance of numerous ferrous alloys [24,25,26,27,28,29,30,31,32,33,34,35,36,37,38,39,40]. Gadag S.P. et al. [38] and Dube D. et al. [39] achieved an increase in cavitation erosion resistance of nodular cast iron and steel by remelting surfaces with a laser beam. Kwok and colleagues conducted similar research on local remelting with a laser beam of stainless steel and laser alloying of surfaces of various alloys [33,36]. Tang C.H, Cheng F.T, and Man H.C. demonstrated that laser remelting of Cu-based alloy surfaces results in improved cavitation erosion resistance [40].

The local surface remelting of parts using the laser beam is an eco-friendly process that provides a strong metallurgical bond between the modified layer and substrate, and the heat-affected zone has a small extent.

The primary goal of this work is to improve cavitation erosion resistance by achieving a fine-grained microstructure in the surface layer without changing the base material’s chemical composition. There is a significant temperature difference between the liquid layer and the solid base as a result of the heat energy that is absorbed in the material during the remelting process [2,3,24]. Additionally, due to convective movements, mixing of the liquid metal occurs. These movements are generated as an effect of the temperature difference between the melted surface and its base and due to the shielding gas blown, as well as the ‘pressure’ of the laser beam. The high temperature gradient causes the liquid metal to solidify quickly once it has melted and mixed. The local surface remelting by laser process offers the advantage of simplicity, flexibility, and reasonable costs, modifying the microstructure without generating a new material [2,25,30,33].

This study investigates the behavior of ultrasonic cavitation erosion on the surface of cast parts made of high-alloy steel, which was locally remelted using the Yb-YAG laser technique. Since its cavitation erosion resistance is relatively low, for the expansion of its application range, a feasible approach involves modifying the surface in areas subjected to pronounced cavitation attack. In this regard, laser beam surface modification has been employed by the authors in this work. The aim followed focuses on the scientific foundation of the mechanism for improving the cavitation resistance of this group of cast high-alloy steels and highlighting the interface reactions between the layer and the substrate, specific to this surface modification technique.

## 2. Materials and Experimental Procedures

The material used in this experimental study is a brand of cast stainless steel with the designation GX40CrNiSi25-20 (W.1.4848), EN 10295 in accordance with the European standard EN 10088, and UNS S31803 in accordance with ASTM A276. Widely utilized in systems operating with cavitating liquids, this material finds applications in diverse fields such as aerospace, automotive, nuclear reactors, marine vessels, civil components, etc.

Table 1 presents the nominal chemical composition of this steel, while Table 2 outlines the guaranteed mechanical characteristic values at room temperature.

**Figure 1:**
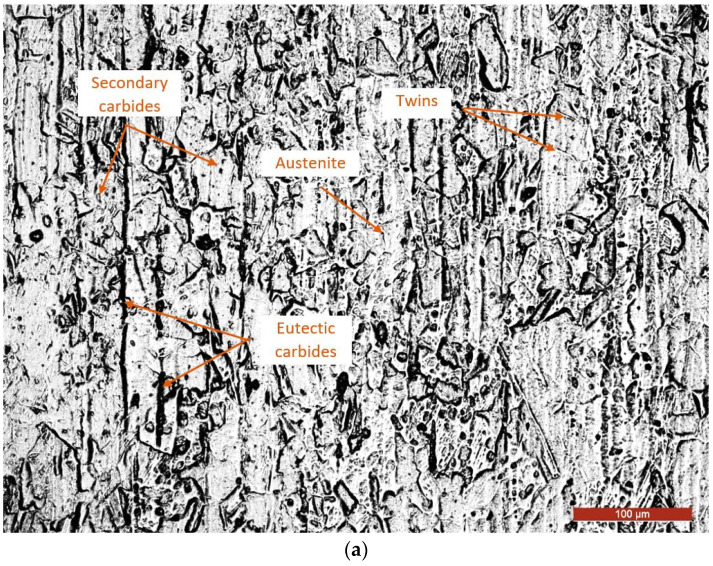

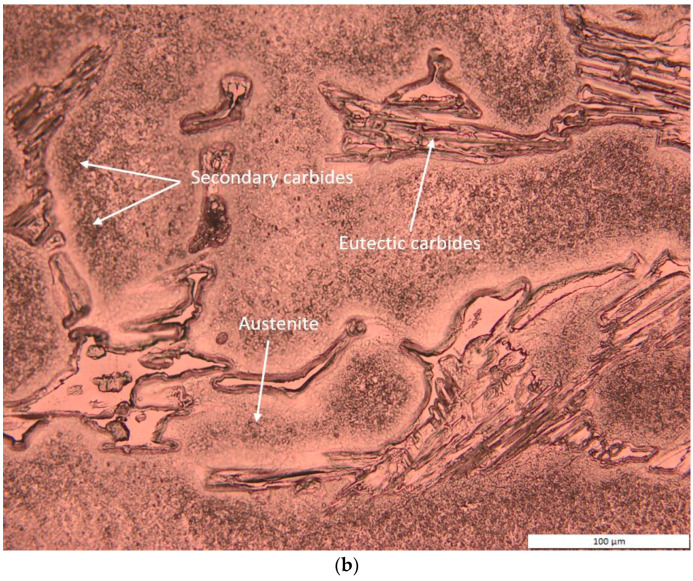
Microstructure of the analyzed steel (etching Villella’s Reagent): (**a**) OM × 200; (**b**) SEM × 1500.

The steel’s microstructure is made up of austenite grains that have recrystallisation twins attached to them, as well as eutectic and secondary carbides orientated towards the former solidification dendrites and on the grain boundaries (Figure 1a,b). These findings are consistent with the findings of other investigations [41,42].

Cast blocks of GX40CrNiSi25-20 steel (W.1.4848), according to EN 10295, were initially cut using disc mills and subsequently processed through milling to create cylindrical samples with dimensions Ø 25 × 40 mm.

High-alloyed steels cast into components often exhibit chemical inhomogeneities due to insufficient diffusion of C, P, S, and alloying elements during the cooling of products in casting molds. To mitigate intradendritic microsegregations, the samples underwent a recrystallization heat treatment for homogenization at T = 1100 °C for 8 h, followed by controlled cooling in an oven at rates of 30–50 °C/h until a temperature below that of the lower critical Ar1 point, and then exposed to air.

**Figure 2:**
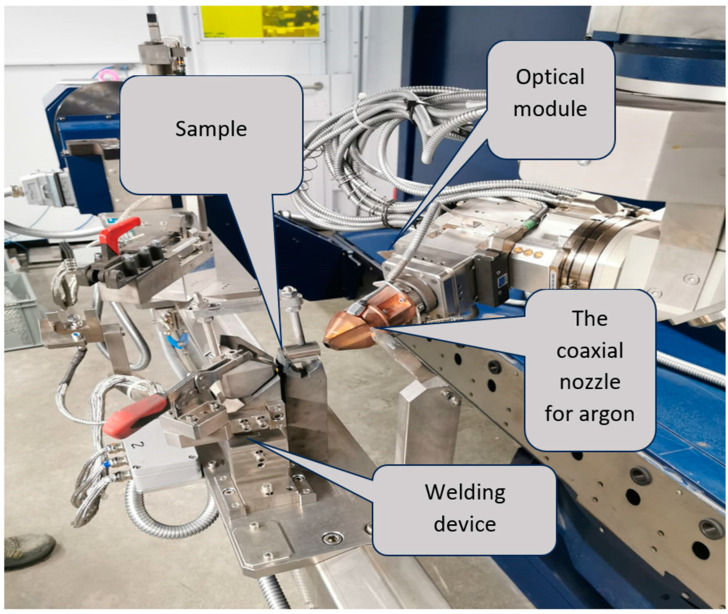
The assembly diagram of the sample in the laser equipment.

Subsequently, some samples were locally remelted on the front surface using a laser beam generated by the ytterbium, Yb-YAG system (Figure 2). The equipment used was of the Trumpf Trucell 7040 type, powered by the Yb-YAG Trudisk 4002 laser source (from TRUMPF Laser SE, Schramberg, Germany) with a laser beam quality of 8 mm × mrad and a wavelength of 1030 nm. Programming for this application was done using the Trutops software provided by Trumpf, version 13.0. The laser beam projection module onto the processed piece is mounted on two numerically controlled linear axes and contains three collimation, reflection, and focusing mirrors arranged on another two rotation axes and one translation axis.

By preliminary experimental trials, the technological laser remelting parameters were selected to obtain a defect-free surface (no porosity, material burn, cracks) suitable for resistance to cavitation erosion. The shielding gas used was argon, with a flow rate of 10 L/min, and the distance from the nozzle to the surface of the processed material sample was maintained at 16 mm. The feed rate was the variable parameter, with values of 0.8 m/min, 1 m/min, and 1.2 m/min. The width of the melted material tracks was 0.9–1 mm, with a 50% overlap, and their depth was 0.3–0.5 mm.

Upon completing the programming and securing the sample in the device, the automated melting process commenced. As the optical module reached the starting position, the laser beam was triggered with a power of 1000 W and a frequency of 5010 Hz in a point projection, with a diameter of 1 mm on the processed sample, resulting in an energy density of 1273 W/mm^2^.

**Figure 3:**
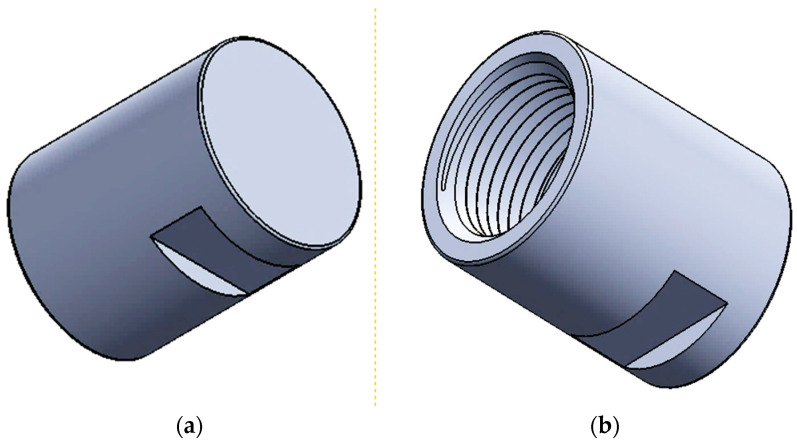

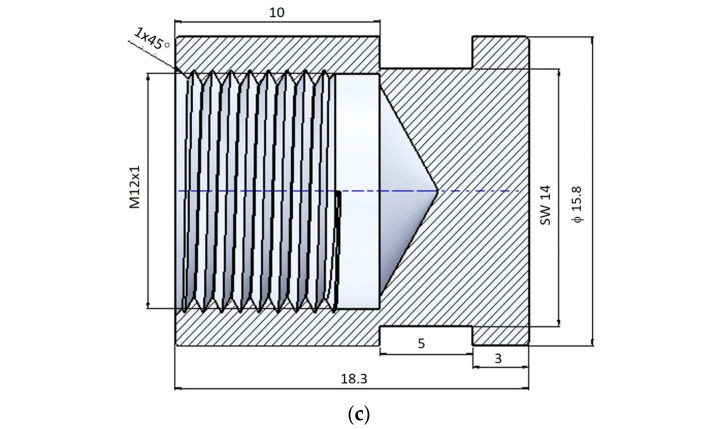
Shape and dimensions of the cavitation samples: (**a**)—cavitated surface; (**b**)—fixing area in the sonotrode; (**c**)—section with the dimensions of the sample.

Following the local surface remelting process, the samples were prepared by machining for cavitation tests. The shape and dimensions of the cavitation samples are shown in Figure 3.

The hardness on the remelted surface was measured under a load of 50 N and a dwell time of 15 s, using the Vickers HVS-10A1 apparatus, from ZwickRoell, Ulm, Germany.

**Figure 4:**
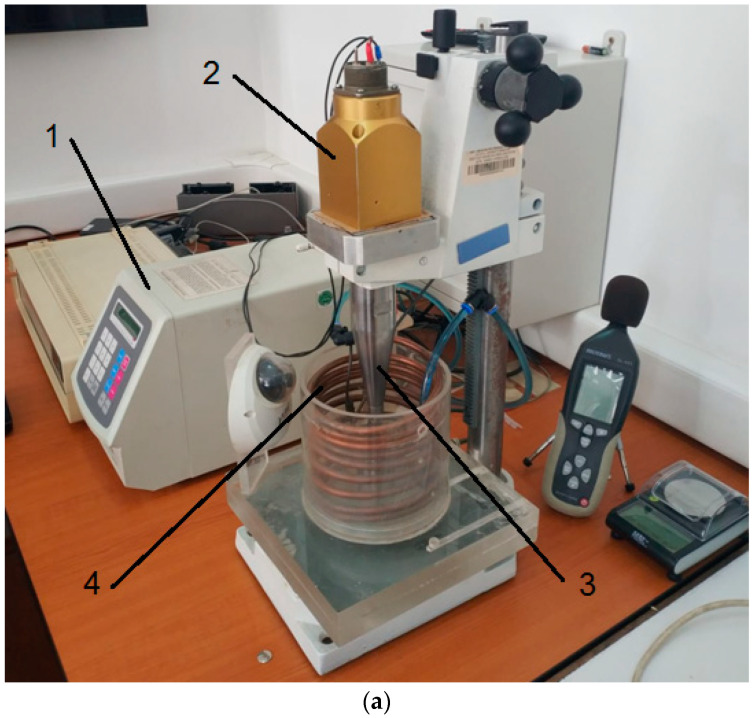

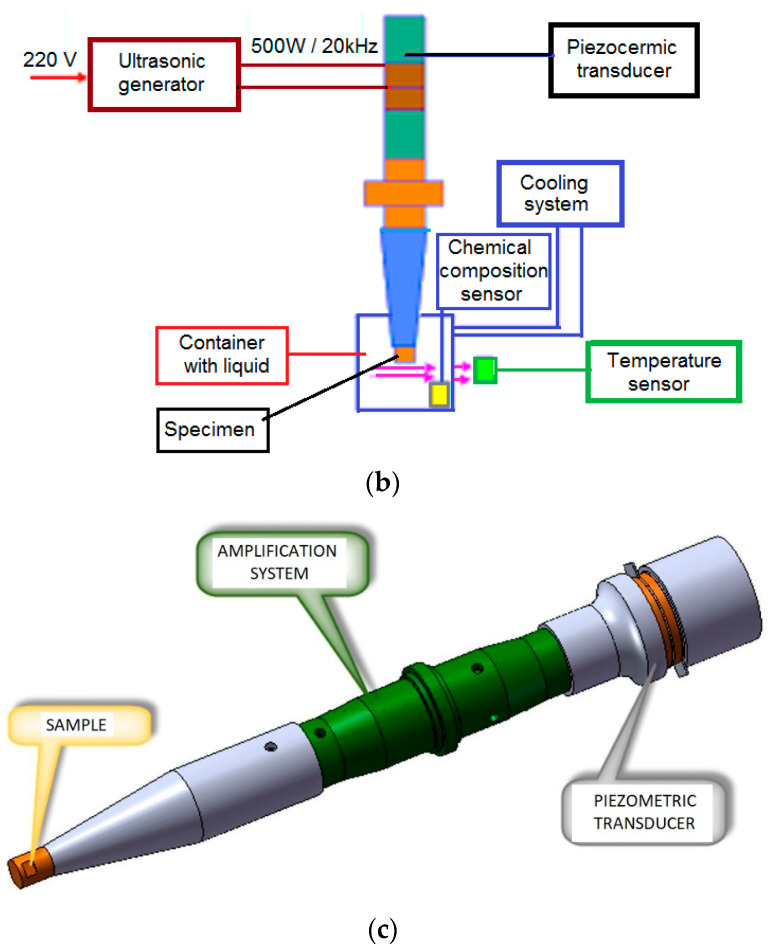
The ultrasonic vibrator apparatus with piezoceramic crystals: (**a**) an overview of the device (1—ultrasonic electronic generator 500 W/20 kHz; 2—piezoceramic transducer 20 kHz; 3—sonothrode; 4—cooling coil); (**b**) the functional diagram of the ultrasonic vibrator apparatus with piezoceramic crystals; (**c**) the mechanical vibration system (piezoceramic transducer with the amplitude amplification system (booster), sonotrode, and cavitation sample).

Surface degradation of the samples through cavitation erosion was induced using the ultrasonic vibrator apparatus, as illustrated in Figure 4. This computer-controlled apparatus is equipped with piezoceramic crystals and software for the automatic control of functional parameters, which is essential in understanding the cavitation hydrodynamic process.

Three samples were examined for each local surface remelting regime. Throughout the investigation, the device’s functional parameters were kept at the ASTM G32-2016 standard’s design values [43].

The working parameters of the equipment were as follows:

- Installed power: 500 W
- Frequency of vibrations: 20 kHz
- Amplitude of vibrations: 50 μm
- Supply voltage: 220 V/50 Hz
- Testing liquid: Tap water
- Temperature of the testing liquid: 22 ± 1 °C

Before initiating the cavitation experiment, the attack surfaces (frontal) were polished to a roughness of Rz = 0.2 ÷ 0.8 μm using a Buehler Phoenix Beta apparatus, Spectrographic Limited, Leeds, UK. Following the laboratory custom at Politehnica University Timisoara, the total cavitation attack duration was 165 min, divided into periods of 5 and 10 min, and 10 periods of 15 min each.

At the end of each testing period, the surfaces eroded by cavitation underwent a cleaning process in tap water, followed by acetone, drying with a stream of hot air, and weighing on an analytical balance with a precision of 10–5 g, type Zatklady Mechaniki Precyzyjnej WP 1. The mass losses were then converted into the mean penetration depth of erosion, MDE, and the mean depth of erosion rate, MDER. The curves representing the experimental values are defined by the relationships [44]:

MDE (t) = A·t·(1−e^−B·t^) (1)

MDER(t) = A·(1−e^−B·t^) + A·B·t·e^−B·t^ (2)

where:

A— it is the statistically determined scale parameter for drawing the approximation/averaging curve, provided that the deviations of the experimental points from it are minimal;
B— it is the shape parameter of the curve.

The topography of the damaged surfaces was examined using the Olympus SYX7 optical stereomicroscope and the TESCAN VEGA 3 LMU scanning electron microscope with Bruker EDX Quantax ((TESCAN VEGA 3 LMU scanning electron microscope with Bruker EDX Quantax from Bruker, Billerica, MA, USA) at both intermediate attack durations and the end of the 165-min period. Subsequently, the samples were longitudinally sectioned and metallographically prepared for examination, both under Leica DM 2700 M optical microscope (Leica Mikrosysteme Vertrieb GmbH, Wetzlar, Germany) and SEM, of the marginal layer where cavitation erosion cracks initiate and propagate.

## 3. Results and Discussion

### 3.1. Hardness Measurements

**Figure 5:**
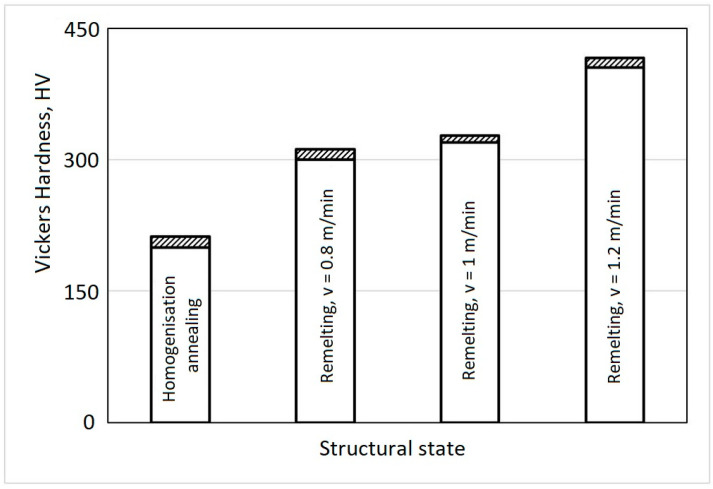
Hardness histogram of base material and locally remelted Yb-YAG samples.

The surfaces remelted with the three values of feed speed underwent grinding-polishing operations and were subsequently subjected to hardness measurements at 8 to 10 measurement points each. The histogram of the hardness values for these remelted surfaces, along with those obtained from the reference material, is depicted in Figure 5.

The analysis of these data reveals a significant increase in material hardness through the Yb-YAG laser process for physical surface modification compared to the specific structural state achieved by homogenization annealing treatment. In the annealed state, the hardness values are approximately 210 HV, while after laser remelting, values of up to approximately 400 HV are attained. For feed speeds of 0.8 m/min. and 1 m/min., the increase in surface hardness is less pronounced, reaching slightly lower values around 310–330 HV. This can be attributed to the microstructural transformations generated by modifying the effective solidification temperature and the degree of undercooling. The laser beam welding process has a very high energy density, on the order of one megawatt per square centimeter (MW/cm^2^), meaning that only a small surface area is affected by heat, while simultaneously providing a large amount of heat with a high cooling rate. The rapid thermal cycle helps to avoid the precipitation of brittle phases. Additionally, a welding speed that is too low leads to excessive melting of the material’s surface, reduces the degree of undercooling, increases the critical radius of crystallization nuclei, the number of nuclei, resulting in a somewhat coarser grain size and microstructure, and lower hardness. Higher welding speeds result in a decrease in the amount of heat introduced into the parts, thereby producing fine microstructures, which lead to an increase in hardness and, consequently, an increase in cavitation resistance. Using feed speeds above 1.2 m/min. resulted in the formation of alternating traces of melted and unmelted material, hindering the achievement of the intended goal.

A hardness increase of 50% to 100% through the application of this surface hardening technique will significantly contribute to improving the cavitation erosion resistance of this high-alloy cast steel.

### 3.2. Specific Curves and Characteristic Parameters of Cavitation Erosion

**Figure 6:**
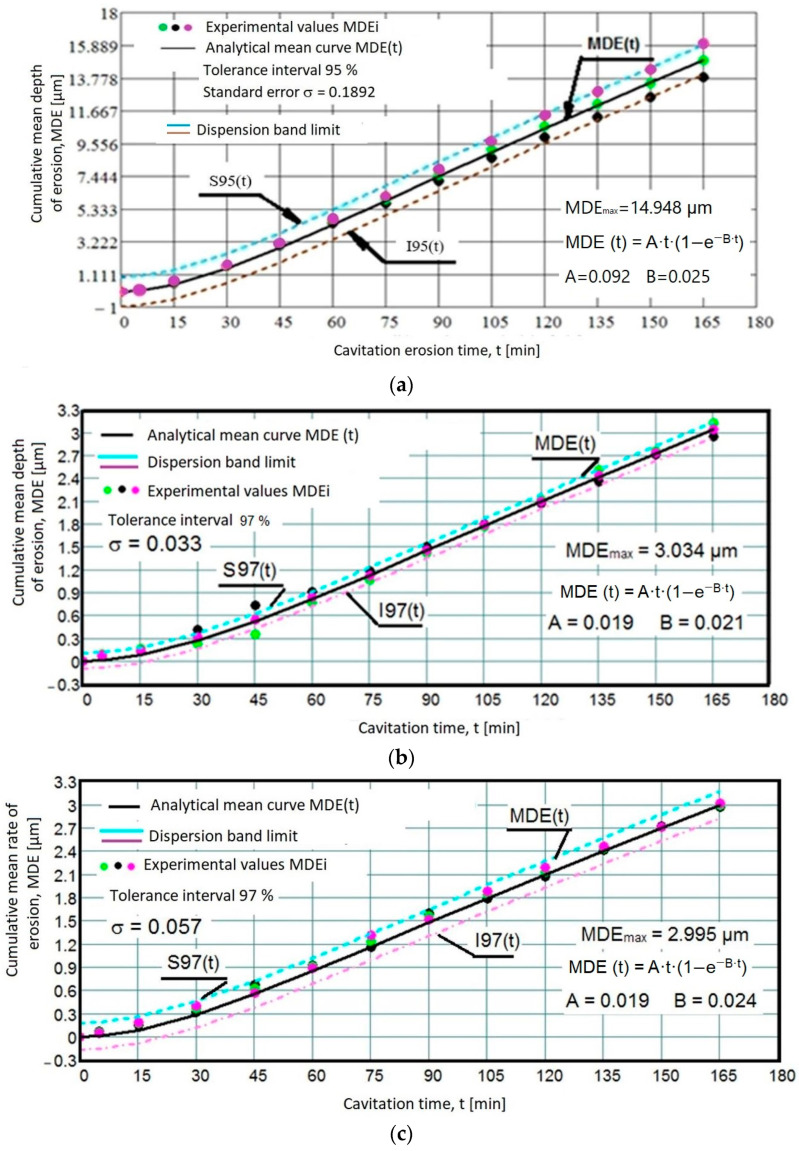

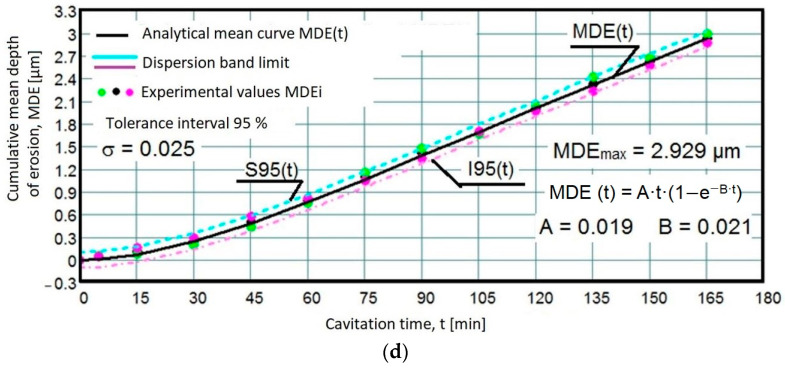
Variation of cumulative mean erosion depth with cavitation attack time. (**a**) initial state of annealing; (**b**) laser remelted state using speed v = 0.8m/min; (**c**) laser remelted state using speed v = 1 m/min; (**d**) laser remelted state using speed v = 1.2 m/min.

To ensure the accuracy of the experiment, Figure 6 illustrates the delineation of the tolerance interval—both upper and lower—representing the range of dispersion for the experimental points. These intervals were calculated using the Mathcad program. The statistical relationships used have the forms:

- the equation of the polynomial regression curve (Equation (1)),
- the standard error of estimation.

(3) $$\sigma_{MDE}={\left[\frac{\sum_{i=0}^{12}{\left(MDE_{i}-MDE(t{)}_{i}\right)}^{2}}{n-1}\right]}^{\frac{1}{2}}$$

where:

- MDE(t)_i_—is the mean penetration depth of erosion defined by the regression curve (Equation (1)) at the time t_i_;
- MDE_i_—is the mean experimental value of the values obtained through the experiment at time t_i_;

(4) $${\mathrm{MDE}}_{i}=\sum_{i=1}^{12}\left(\frac{4\cdot {\Delta m}_{i}}{\rho \cdot \pi \cdot d_{p}^{2}}\right)$$

where: Δm_i_—is the mass of material lost by the tested sample during the time interval corresponding to the intermediate period “i,” which ends at the cumulative time t_i_ (for example, for i = 0, t_0_ = 0, for i = 1, the duration is t_1_ = 5 min, for i = 2, the duration is t_2_ = 15 min, and from i = 3 to i = 12, the duration t_i_ increases by 15 min).

The dispersions of experimental values, compared to the mediating curves and within tolerance intervals, serve as an expression of the accuracy of the cavitation tests on each set of three samples, regardless of their structural state. The standard deviation values (ranging from 0.025 to 0.1892) indicate that the test was conducted correctly, procedural steps were followed, and the parameters of the vibrator apparatus, which determined the destructive intensity of cavitation, were well controlled.

**Figure 7:**
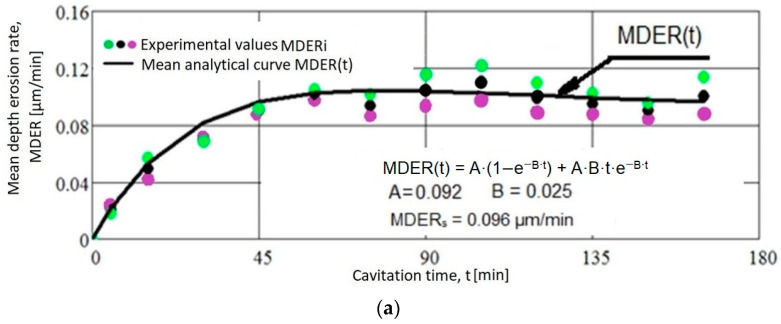

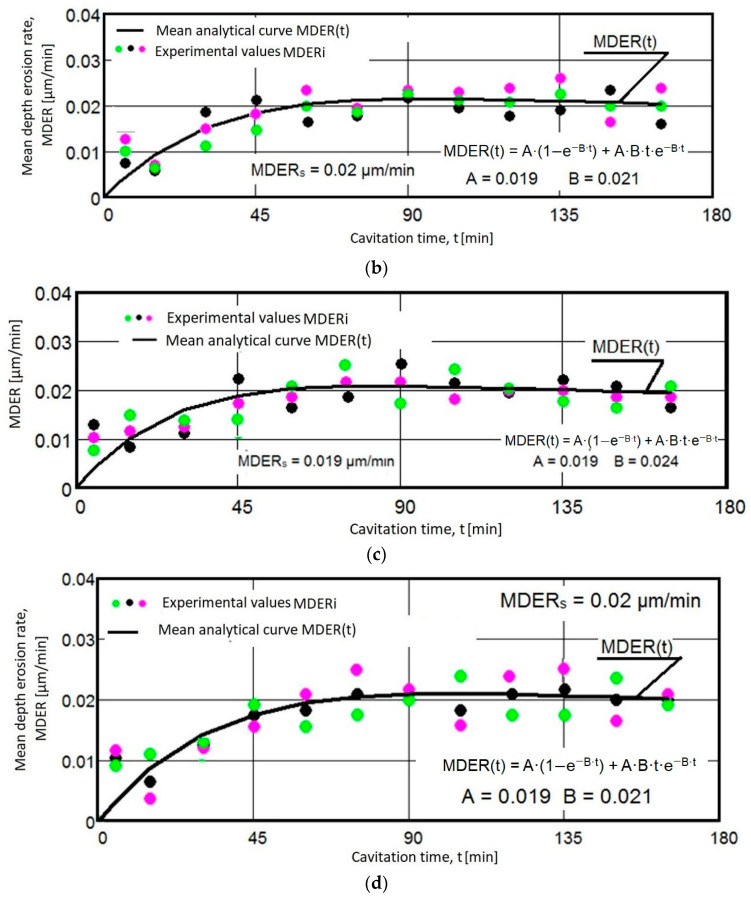
Variation of mean erosion penetration rate with the cavitation attack time. (**a**) annealed state; (**b**) laser remelted state using speed v = 0.8m/min; (**c**) laser remelted state using speed v = 1 m/min; (**d**) laser remelted state using speed v = 1.2 m/min.

The data presented in the diagrams in Figure 6 underscore the following aspects:

- The high accuracy of measurements on sets of three samples, regardless of their structural state, is reflected by the standard deviation values (0.025…0.1892) and the dispersion band of values corresponding to tolerance intervals of (95–97%).
- The test was conducted accurately, procedural steps were diligently followed, and the vibrator apparatus parameters, determining the destructive intensity of cavitation, were well controlled throughout the experiment.
- Natural differences between the values of standard deviation suggest that laser remelting with a speed of v = 1.2 m/min leads to obtaining a microstructure that provides the best resistance to cavitation erosion. Previous experiences [13,17,21,22] suggest that the dispersion of experimental values results from refining the microstructure and enhancing the mechanical properties of surfaces exposed to vibrating cavitation erosion.
- A close relationship exists between the differences among the three experimental values obtained on each set of samples at the same duration of cavitation attack and the dimensions (or mass) of the grains ejected in the intermediate cavitation intervals. These differences are well highlighted in the variations of the experimental values, averaged algebraically, compared to the MDER(t) mean curves in Figure 7.

The diagrams shown in Figure 7 display the experimental values of the mean erosion penetration rates related to the intermediate cavitation intervals and the MDER(t) mediation curves.

The values of the ratio between the maximum values of the mean erosion depths, MDEmax and between the mean erosion penetration rates, MDERs of the three sets of three samples, are as follows:

$$\frac{MDEmax1}{MDEmax3}=1.035\;\;\;\;\;\;\;\;\;\;\;\;\;\;\;\;\frac{MDERs1}{MDERs3}=1.52$$

$$\frac{MDEmax2}{MDEmax3}=1.022\;\;\;\;\;\;\;\;\;\;\;\;\;\;\;\;\frac{MDERs2}{MDERs3}=1.052$$

$$\frac{MDEmax1}{MDEmax2}=1.013\;\;\;\;\;\;\;\;\;\;\;\;\;\;\;\;\frac{MDERs1}{MDERs2}=1.0$$

The results demonstrate slight differences in cavitation resistance. Specifically:

- The increase in strength achieved by remelting at a speed of 1.2 m/min, compared to samples where laser remelting speeds of 0.8 m/min and 1.0 m/min were used, is 1.035 times higher when analyzing the maximum value of the mean erosion depth (MDEmax) and 1.052 times higher when analyzing the MDERs value towards which the mean penetration depth of erosion rate tends to stabilize.
- The increase in resistance achieved at a working speed of 1.2 m/min, compared to samples where a laser remelting speed of 1 m/min was used, is higher, 1.022 times, when analyzing the maximum value of the mean depth of erosion (MDEmax) and of the same level after the MDERs value, towards which the mean penetration depth of erosion rate tends to stabilize.
- Between the resistances of the surfaces remelted with the laser beam at speeds of 0.8 m/min and 1.0 m/min, there is a negligible difference in resistance to cavitational stresses of 1.013 times, as per the maximum value of the mean erosion depth (MDEmax).

**Figure 8:**
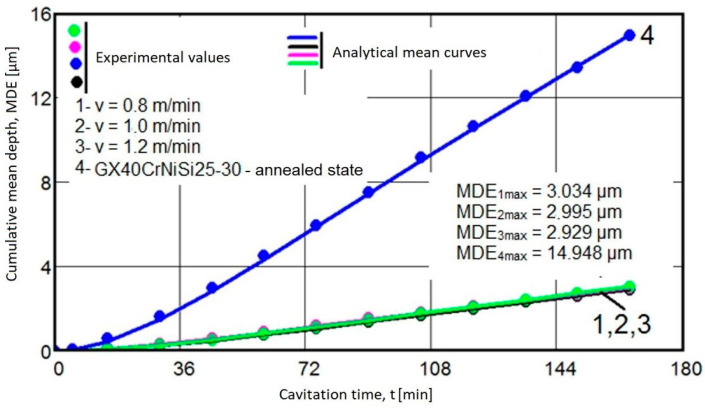
The variation of the mean erosion depth, MDE, with the cavitational attack time: 1—remelting with v = 0.8 m/min, 2—remelting with v = 1 m/min, 3—remelting with v = 1.2 m/min, 4—annealed state.

**Figure 9:**
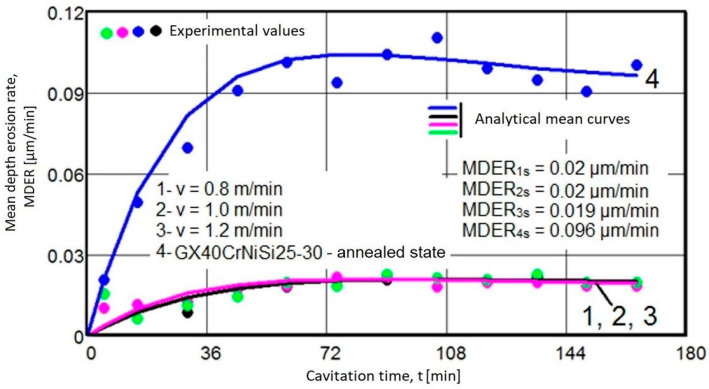
The variation of the mean penetration depth of erosion rate, MDER, with the cavitation attack time, 1—remelting with v = 0.8 m/min., 2—remelting with v = 1 m/min., 3—remelting with v = 1.2 m/min., 4—annealed.

Using the algebraic mean values of the experimental values acquired on sets of three samples each (per Figure 6 and Figure 7), the specific curves MDE(t) and MDER(t) are comparatively given for the four structural states in the graphs from Figure 8 and Figure 9.

The data from the two diagrams allow the following observations:

The evolution of the mediating curves and the level of the experimental values, algebraic means, indicate that the behavior of surfaces hardened by laser remelting is significantly superior to that achieved through the volumic annealing treatment for homogenization.

The dispersions of experimental values obtained on samples hardened by laser remelting are much smaller than those obtained with annealing for homogenization, as indicated by the values of standard deviations (σ).

In terms of structural resistance to cavitation microjet stresses, the increase achieved through laser remelting, regardless of the remelting speed, compared to the annealed state, ranges from 4.92 to 5.1 times, according to the maximum values of the cumulative mean depths (MDEmax) defined by the mediating curves. Additionally, it ranges from 4.8 to 5 times, according to the average erosion penetration rate values (MDERs) at the end of the test, known as the value toward which the MDER(t) curve asymptotically tends at the end of the total cavitation duration.

According to the ASTM G32-2016 standard, the inverse of the erosion penetration depth rate over the stabilization period defines the cavitation resistance, R_cav_. As a result, the report:

$$R_{\mathrm{cav}}(v=0.8m/\min.)=\frac{1}{0.02}=50$$

$$R_{\mathrm{cav}}(v=1m/\min.)=\frac{1}{0.02}=50$$

$$R_{\mathrm{cav}}(v=1.2m/\min.)=\frac{1}{\;0.019}=52$$

$$R_{\mathrm{cav}}(\mathrm{annealing})=\frac{1}{0.096}=10.41$$

In conclusion, the surface modification technique causes a substantial increase in cavitation erosion resistance, approximately 4.8 to 5 times, compared to the initial state subjected to homogenization annealing treatment.

### 3.3. Structural Modifications

**Figure 10:**
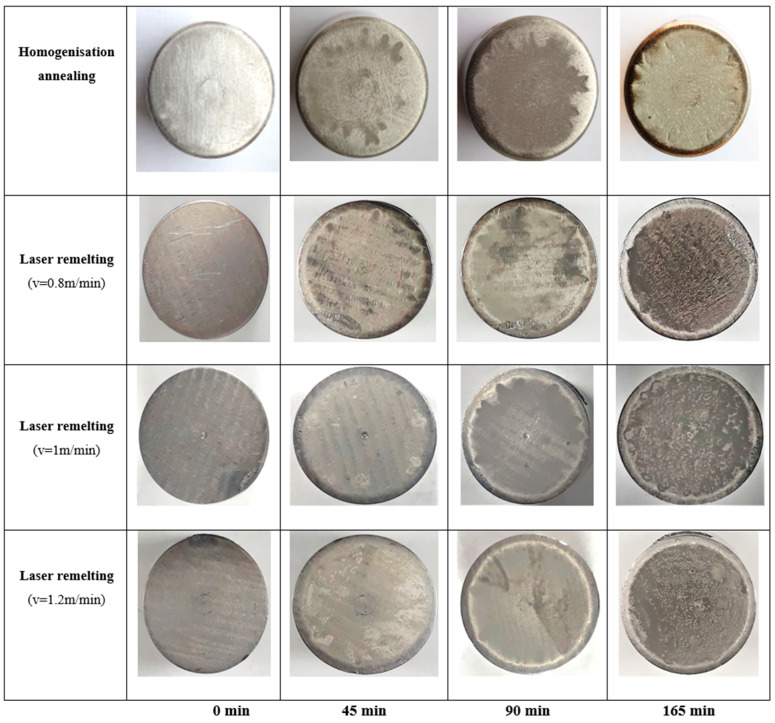
Macrography of the surfaces exposed to cavitation at variable durations of time.

After every cavitation attack period, the progression of the cavitation phenomena was recorded by taking surface photos of the samples with a Canon PowerShot SX200 IS camera (Figure 10). Analysis of these images reveals the following observations:

- In the reference state, during the first 30 min of cavitation attack, there is no noticeable deterioration of the surface. However, with a longer duration, a continuous increase in the density of pinches in the material and their depth is observed.
- With an increase in cavitation attack duration, the surface structure resulting from the laser remelting regime with a speed of v = 0.8 m/min is slightly more affected, indicating that this regime leads to lower resistance to surface degradation.
- The best resistance to vibratory cavitation attack is obtained by the structure resulting from the laser remelting regime with a speed of v = 1.2 m/min, with the evolution of destruction in terms of area and depth being significantly inferior to the other two regimes.
- These images of erosion evolution highlight that the resistance of a laser-remelted surface is dependent on the process parameters, namely the laser remelting speed.
- The photographic images in Figure 10 align with the results provided by the specific cavitation curves in Figure 8 and Figure 9.

Compared to images of surfaces subjected to cavitation attack in the case of GX40CrNiSi25-20 steel in its initial state of homogenizing annealing, finer grain finish and, implicitly, microstructure improvement are observed on surfaces remelted with laser technology.

**Figure 11:**
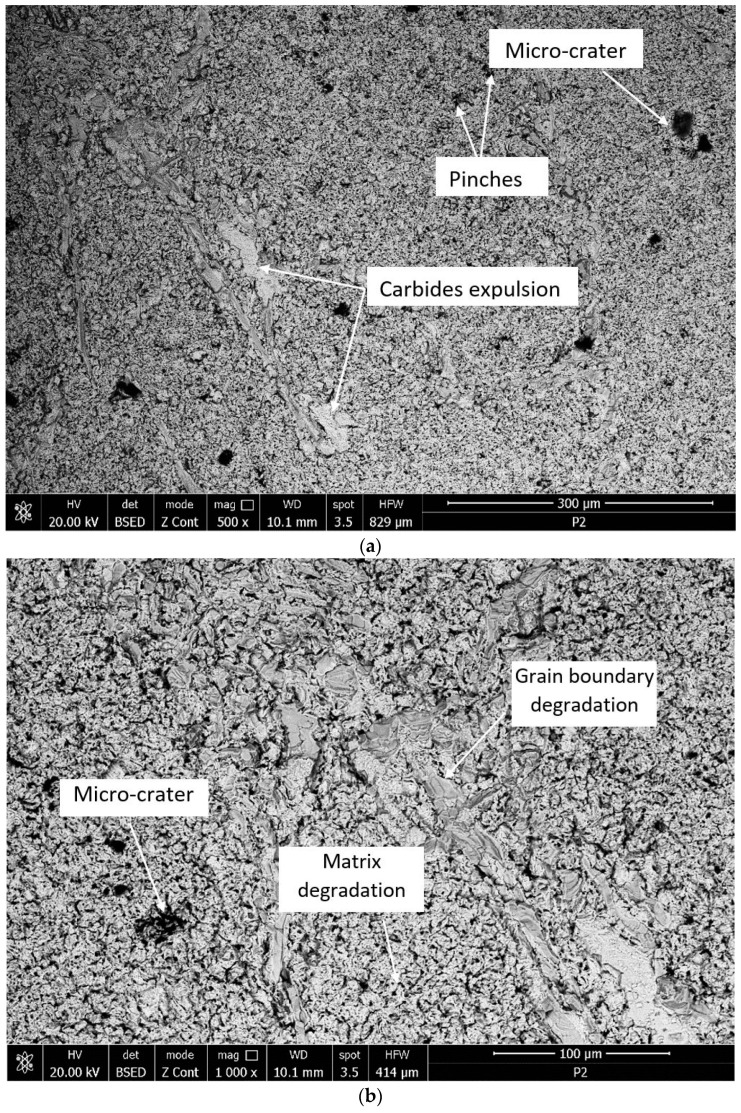
Microfractographic image of the reference metal surface (annealed state) after the 165-min cavitation test: (**a**)—×500; (**b**)—×1000.

**Figure 12:**
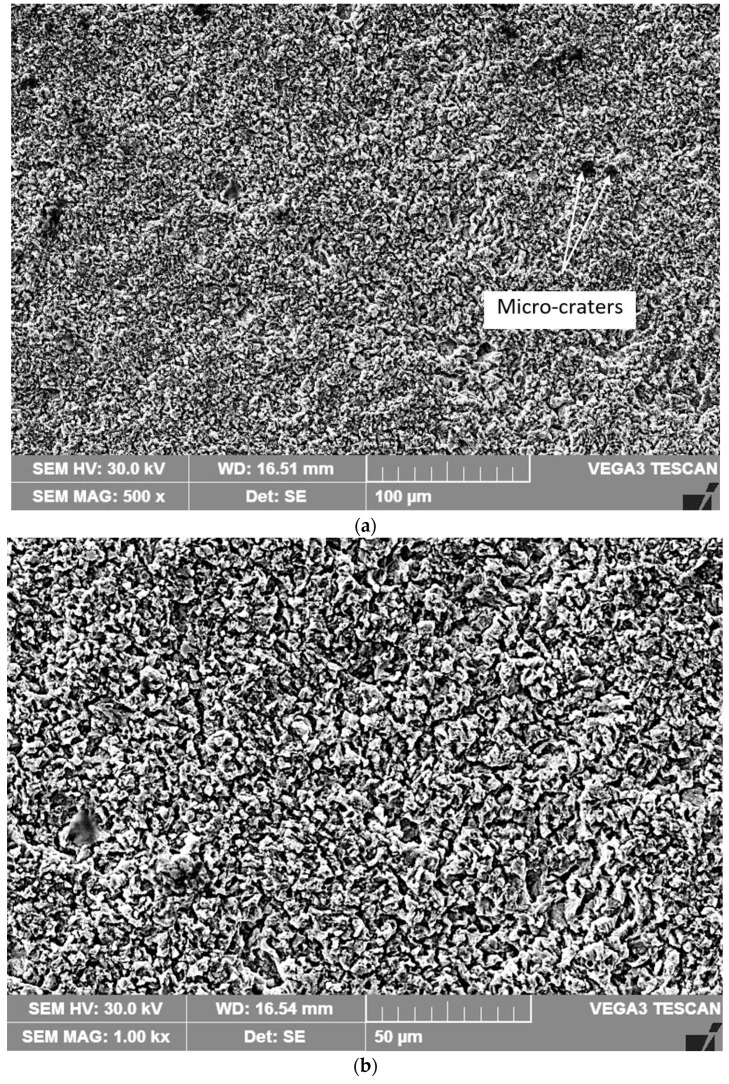
Microfractographic image of the laser remelted surface after the 165-min cavitation test: (**a**)—×500; (**b**)—×1000.

The microfractographic investigations performed on the surfaces of the base material and on the laser-remelted ones (Figure 11 and Figure 12) after the 165-min cavitation test highlight the differences in the behavior of the material in the two structural states.

As a rule, the initiation of erosion through cavitation takes place on the boundaries of the austenite grains by removing the carbide particles, after which an extension of this phenomenon is found inside these grains. Although in both situations, the appearance of craters caused by the preferential erosion of the boundaries between the grains is evident, their shape and dimensions are different. Resulting from the high fragility of the eutectic carbide particles in the base metal, the craters formed as a result of their expulsion and the partial or complete pulling out of some crystalline grains are much deeper (over 32 µm) compared to those resulting from surface erosion remelted (maximum depth does not exceed 9 µm).

The microcavitation craters present on the laser-remelted surface represent the places of the former secondary carbide particles of the alloying elements. At the same time, it is observed that the degradation of the reference material surface is irregular, and that specific to the laser-remelted surface is uniform (Figure 11 compared to Figure 12). These results are fully confirmed by those presented in other research papers on cavitation erosion of austenitic stainless steels [3,4,7].

**Figure 13:**
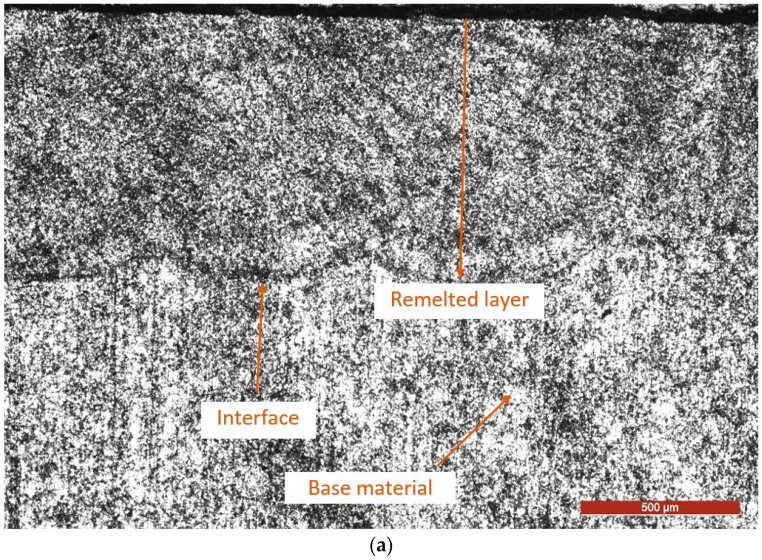

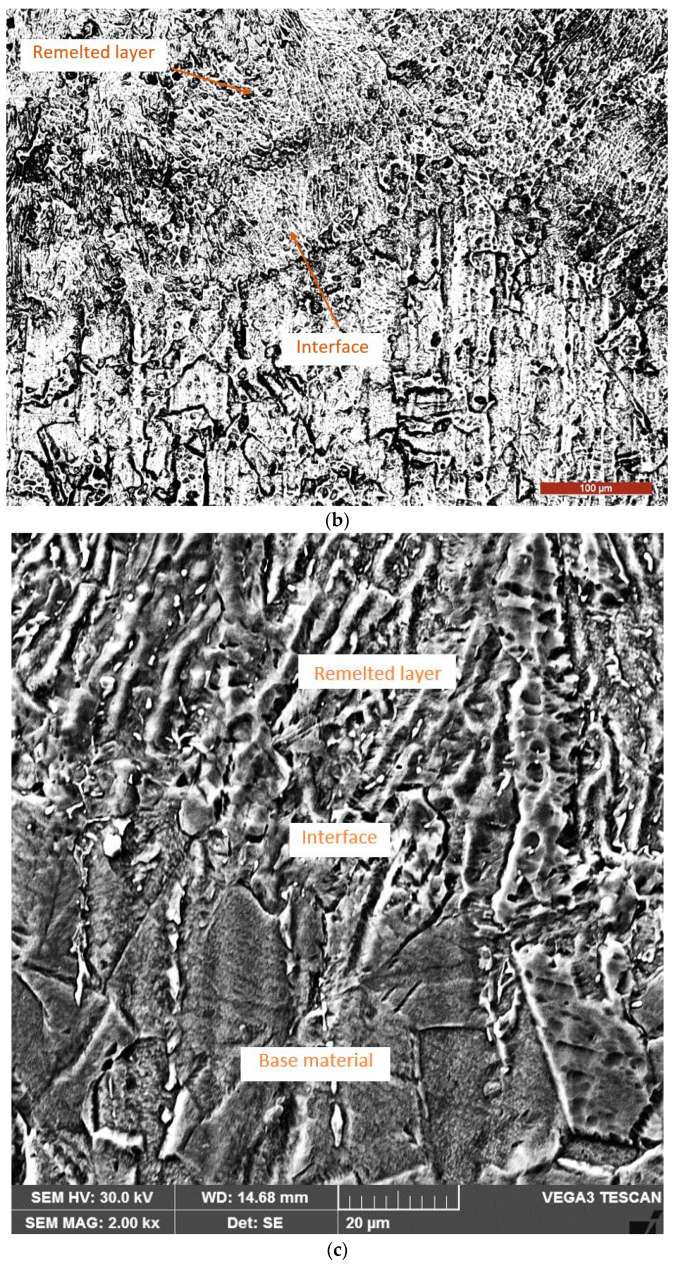
The microscopic image of the laser remelted layer—substrate system after the cavitation test for 165 min: (**a**)—OM × 50; (**b**)—OM × 200; (**c**)—SEM × 2000.

Microscopic examination of cross-sections through the surface layers that were remelted and cavitation tested for 165 min. (Figure 13) proves that the laser process is manifested by the accentuated finishing of the granulation and of the carbide particles precipitated both from the liquid phase and from the austenite. The relatively high forward speeds of the laser beam make the heat cycle faster, the degree of subcooling higher, the critical radius of the crystallization seed smaller, and the obtained microscopic structure finer. However, the finer the granulation, the greater the resistance to dynamic shock stresses generated by the cavitation bubbles and, implicitly, the higher the resistance to cavitation erosion.

At the layer-substrate interface, no metal continuity defects such as porosity, cracks, or seams are observed. The connection between these two components of the system is of a metallurgical nature. The microstructure of the interface consists of grains of γ solid solution (austenite) with an equiaxial shape, having small dimensions and uniformly distributed carbide particles, mainly inside them.

## 4. Conclusions

The local surface remelting by the laser process of the samples from the investigated alloy results in an increase in hardness from approximately 200–210 HV in the annealed state to values of approximately 380–390 HV, depending on the linear energy introduced into the material. This rise in hardness significantly contributes to the improvement of resistance to cavitation erosion.

The surface exhibiting the highest resistance to vibrating cavitation erosion is the one for which the melting laser speed is v = 1.2 m/min. The increase in resistance, compared to the other two regimes, is as follows:

- According to the value of the MDEmax parameter, the achieved increase is 2.2% compared to the regime with v = 1 m/min and 3.5% compared to the structure obtained by using the laser melting speed v = 0.8 m/min.
- According to the value of the MDERs parameter, the increase achieved is 5.2% compared to the structure obtained by using laser melting speeds v = 0.8 m/min and v = 1 m/min.
- Compared to the initial reference state of the material, the laser surface modification technique produces an increase in resistance to cavitation attack from 4.9 times to 5.1 times after the values of the cumulative mean depths, MDEmax, respectively from 4.8 times to 5 times, according to the values of the mean penetration depth of erosion rate, MDERs.

The investigations conducted using the optical microscope and the scanning electron microscope have demonstrated that, for the laser processing parameters employed, a modified layer with a thickness of several hundred micrometers has formed on the GX40CrNiSi25-20 substrate. The layer shows no discontinuities in metallic continuity (cracks and pores) and is strongly bonded to the substrate through a resistant interface. The changed layer has a fine grain structure and a homogenous microstructure.

One can conclude that laser surface remelting of GX40CrNiSi25-20 substrate had a positive influence regarding the cavitation erosion phenomenon.

## Figures and Tables

**Table 1:** GX40CrNiSi25-20 steel chemical composition.

Carbon (C)	0.38%
Silicon (Si)	1.62%
Manganese (Mn)	1.49%
Chromium (Cr)	25.20%
Nickel (Ni)	20.80%
Molybdenum (Mo)	0.34%
Sulfur (S)	0.027%
Phosphorous (P)	0.031%
Iron (Fe)	Balance

**Table 2:** Mechanical properties of GX40CrNiSi25-20 steel at room temperature.

Yield strength, Rp_0,2_ [N/mm^2^]	234
Ultimate tensile strength, Rm [N/mm^2^]	452
Elongation at break, A5 [%]	7.1
Necking, Z [%]	36
Hardness, HV [daN/mm^2^]	198

## Data Availability

Data is contained within the article.
